# A Click Chemistry‐Based Proteomic Approach Reveals that 1,2,4‐Trioxolane and Artemisinin Antimalarials Share a Common Protein Alkylation Profile

**DOI:** 10.1002/anie.201512062

**Published:** 2016-04-18

**Authors:** Hanafy M. Ismail, Victoria E. Barton, Matthew Panchana, Sitthivut Charoensutthivarakul, Giancarlo A. Biagini, Stephen A. Ward, Paul M. O'Neill

**Affiliations:** ^1^ Department of ChemistryUniversity of LiverpoolLiverpoolL69 7ZDUK; ^2^ Research Centre for Drugs and DiagnosticsLiverpool School of Tropical MedicinePembroke PlaceLiverpoolL3 5QAUK

**Keywords:** antimalarial, artemisinin, chemical biology, probes

## Abstract

In spite of the recent increase in endoperoxide antimalarials under development, it remains unclear if all these chemotypes share a common mechanism of action. This is important since it will influence cross‐resistance risks between the different classes. Here we investigate this proposition using novel clickable 1,2,4‐trioxolane activity based protein‐profiling probes (ABPPs). ABPPs with potent antimalarial activity were able to alkylate protein target(s) within the asexual erythrocytic stage of Plasmodium falciparum (3D7). Importantly, comparison of the alkylation fingerprint with that generated from an artemisinin ABPP equivalent confirms a highly conserved alkylation profile, with both endoperoxide classes targeting proteins in the glycolytic, hemoglobin degradation, antioxidant defence, protein synthesis and protein stress pathways, essential biological processes for plasmodial survival. The alkylation signatures of the two chemotypes show significant overlap (ca. 90 %) both qualitatively and semi‐quantitatively, suggesting a common mechanism of action that raises concerns about potential cross‐resistance liabilities.

Despite concerns about the recent emergence of drug‐resistance,^1^ the artemisinins (**1 a**–**c**, Figure 1) remain frontline agents for the treatment of malaria.^2^ Understanding the mechanism of action of such an important class has been the subject of intense research over the last two decades.^2^ The proposed mechanism of bioactivation of the class involves the cleavage of the endoperoxide bridge by a source of Fe^2+^ or heme. This cleavage results in the formation of oxy‐radicals that rearrange into primary or secondary carbon centered radicals (or electrophilic carbocations through single‐electron transfer oxidation) (Scheme 1).^3^

**Figure 1:**
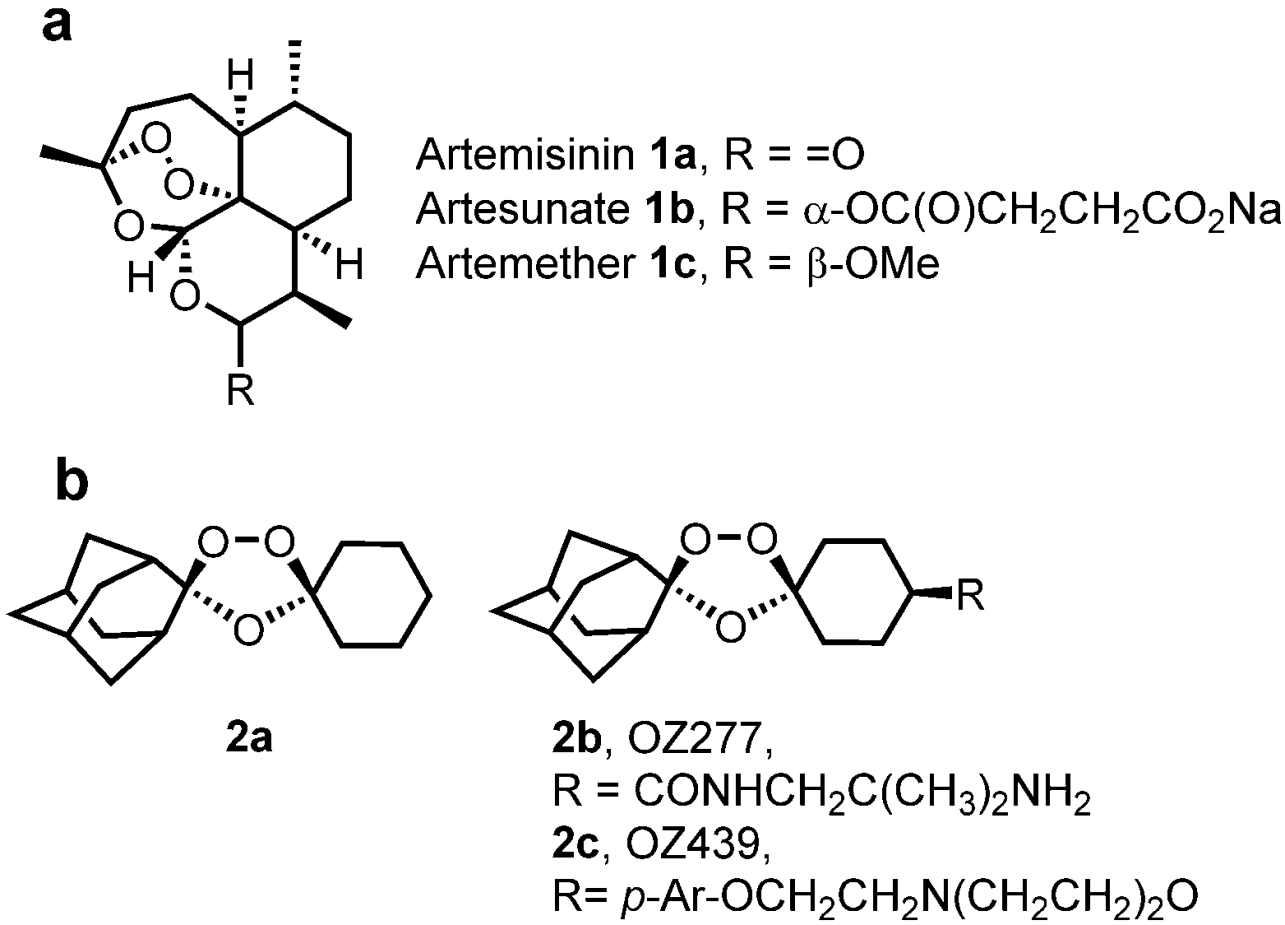
Artemisinin **1 a**, semi‐synthetics **1 b** and **1 c** with structures of frontline synthetic peroxide based antimalarials OZ277 (**2 b**) and OZ439 (**2 c**).

**Scheme 1:**
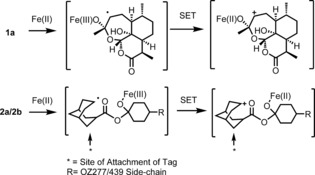
Iron‐mediated fragmentation of endoperoxides to reactive intermediates capable of reacting with parasite proteins. (Only the secondary carbon‐centered radical derived from artemisinin is depicted.)

These reactive intermediates are proposed to alkylate proteins and form adducts with essential parasite macromolecules that result in the rapid death of the parasite. However, the detail of these important alkylation reactions are sparse and the underlying hypothesis remains controversial.^2^

The debate has broadened with the development of highly active fully synthetic endoperoxides based on the pharmacophore of artemisinin namely the trioxolanes (**2 a**)^4^ and the tetraoxanes.^5^ From the perspective of the underlying chemical mechanism of activation of peroxides and from a cross‐resistance risk it is important to establish if these different endoperoxide chemotypes share a common mechanism of action or not.

A study of 1,2,4‐trioxolanes using monoclonal antibodies has demonstrated parasite protein alkylation with both OZ277 (**2 b**) and OZ439 (**2 c**).^6^ However, the methods employed in this work were unable to definitively identify the targeted proteins.

In a recent study, Wang et al.^7^ used a non‐optimised ART‐alkyne activity‐based protein‐profiling probe that via click‐chemistry reactions was associated with some 124 *P. falciparum* proteins.^7^ In this study, a further 125 proteins are reported as being identified in single replicate experiments only, raising concerns of the specificity of the approach.^7^ Concurrently, using both ART‐alkyne and azide optimized probes we were able to identify 59 *P. falciparum* proteins with high confidence that were specifically alkylated by ART pointing towards a pleiotropic mechanism of drug action.^9^ In our study we deployed probes with reduced linker length and lipophilicity compared to the probe used by Wang et al., and we used both copper‐dependent and copper‐free reactions over a shortened incubation time (optimized to 1 h as originally described,^8^ cf. Wang et al., reaction time of 3 h) together with control non‐peroxide probe partner equivalents. These methodological differences help to improve the specificity and pharmacological relevance of the alkylated proteins identified. Significantly in our study only 6 proteins were identified in less than two replicate experiments (cf. 125 in Ref. 7) with an azide probe, demonstrating the improved specificity of our approach using optimized active and control probe based methodology.

Here, using our refined approach, we describe the rational design of potent activity‐based protein‐profiling probes (ABPPs) based on a 1,2,4‐trioxolane antimalarial core in order to characterize their malaria parasite protein alkylation fingerprint. Significantly, we show that these synthetic 1,2,4‐trioxolanes and a semi‐synthetic ART share an overlapping parasite protein‐alkylation signature suggestive of a common mechanism of action for the endoperoxide class of antimalarial.

The probes were designed with the alkyne/azide click handle sited within the adamantane ring system since this is the site (Scheme 1) of reactive C‐radical/carbocation generation post activation by Fe^II^. We also deployed a bio‐orthogonal copper‐free “click” methodology via the use of an azide analogue along with its negative control Figure 2. The azide probes were included in our analysis to demonstrate that the protein alkylation was solely due to iron mediated activation with no role for the copper in peroxide activation during sample work‐up as discussed previously.^9^ This Cu‐free click reaction possesses comparable kinetics to the Cu‐catalyzed reaction and proceeds within minutes in live cells with no apparent cytotoxicity issues.8b The complementary reporter tags used in our study can either be sourced commercially or synthesized by literature procedures (Figure S1 in the Supporting Information).

**Figure 2:**
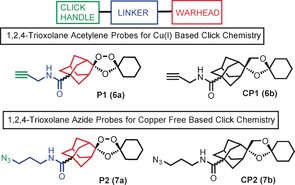
Rational design of endoperoxide activity based probes **P1** (**6 a**) and **P2** (**7 a**) with structures of control non‐peroxidic derivatives **CP1** (**6 b**) and **CP2** (**7 b**).

Scheme 2 a provides an overview for the synthesis of alkyne probe **P1** (**6 a**) and azide probe **P2** (**7 a**) along with control probes **CP1** (**6 b**) and **CP2** (**13 b**). The first step in the synthesis of the trioxolane probes involved the Koch–Haaf carbonylation of hydroxyl adamantanone to give the methyl ester **3**. Co‐ozonolysis of oxime **4** with **3** provided trioxolane **5**; hydrolysis of the methylester function of **5** followed by EDC‐mediated coupling of propargyl amine provided probe **P1** (**6 a**) in good overall yield. Coupling of 3‐azido‐1‐propylamine to **6** provided the azide probe **P2** (**7 a**) as shown in Scheme 1. The control probe **CP1** (**6 b**) was made by a similar approach using diol **9** in a cyclisation reaction with **3** to produce the corresponding carba ester analogue **10**. Hydrolysis of **10** and coupling of the resultant acid with propargyl amine afforded **6 b** in good yield (70 %). Azide **7 b** was made in a similar manner by hydrolysis and coupling as shown in Scheme 2 b.

**Scheme 2:**
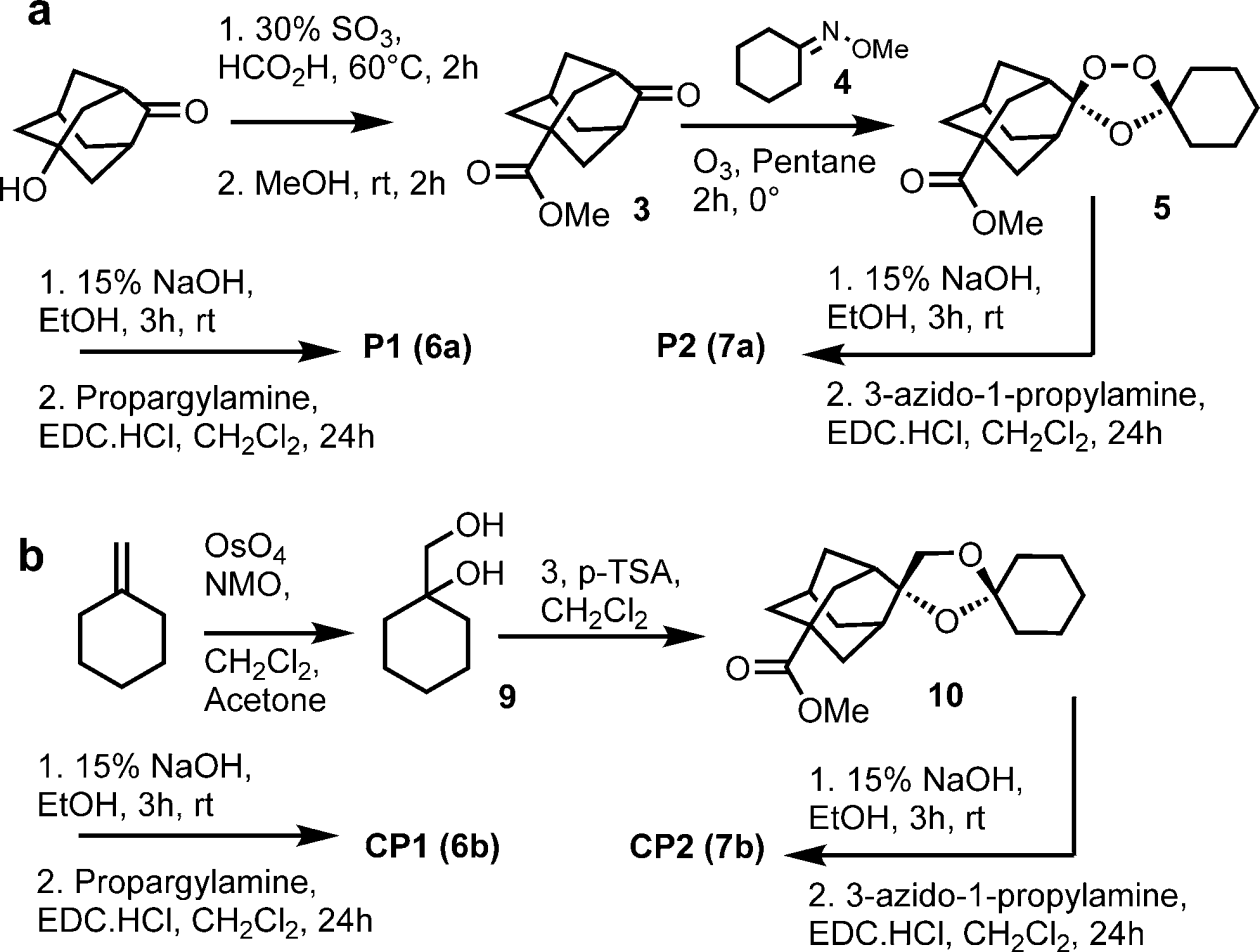
Synthesis of probes and control molecules used in chemical proteomic interrogation of drug activation.

The active probes containing azide/alkyne functionality retained potent antimalarial activity as determined by their IC_50_ in vitro against *P. falciparum* 3D7 parasites (Figure S2 and Table S1). The non‐peroxidic negative control probes CP1 (**6 b**) and CP2 (**7 b**) had no appreciable activity (IC_50_ >10 μm) in these assays,^10^ confirming the essentiality of the endoperoxide‐bridge for drug activity and further validating our probe plus control pair strategy for biologically relevant target protein identification.

As a next step, in vitro cultures of *P. falciparum* 3D7 were incubated with 1 μm of the alkyne trioxolane based probe **P1** (**6 a**) or its corresponding control **CP1** (**6 b**), for 6 hours, a time already shown to be pharmacologically relevant, causing irreversible parasite toxicity.^7^, ^9^, ^10^ Following incubation with **P1** (**6 a**) alkylated proteins were extracted from erythrocyte free parasites and tagged with Alexa Fluor 488 azide via a click reaction. This was processed for 1D‐Gel analysis as described in the supporting information section. After fluorescence imaging of the 1D‐Gel, the strongest labeling was observed in the region of 12–75 KDa (Figure S2 c). Importantly, labeling was not observed for the corresponding “negative control” probe **CP1** (**6 b**) samples. Additional experiments were carried out to investigate the lowest concentration of reporter azide required to obtain maximum labeling with minimum background on SDS gels (data not shown). As depicted in Figure S2 c, the Alexa Fluor 488 azide at a concentration of 20 μm was able to distinguish the **P1** (**6 a**) labeling profile from its corresponding control **CP1** (**6 b**).

To rule out the possibility that Cu^I^ may have led to parasite independent (artifact) protein alkylation and to further validate the results a more stringent bio‐orthogonal copper‐free “click” methodology was employed using cyclooctyne reporter tags as depicted in Figure 3.8b Replacement of an alkyne with an azide group in **P2** (**7 a**) had no detrimental effect on the antimalarial potency of the trioxolane azide probe as indicated in Figure S2, Figure 3 and Table S1. However, the labeling profile intensity with **P2** (**7 a**) was much higher compared to **P1** (**6 a**) (Figure 3 c,d) suggesting greater efficiency of the copper‐free click reaction.8b Reduction of P2 (**7 a**) concentrations to 100 nm (LC90) had no impact on the labeling pattern observed (Figure 3 f).

**Figure 3:**
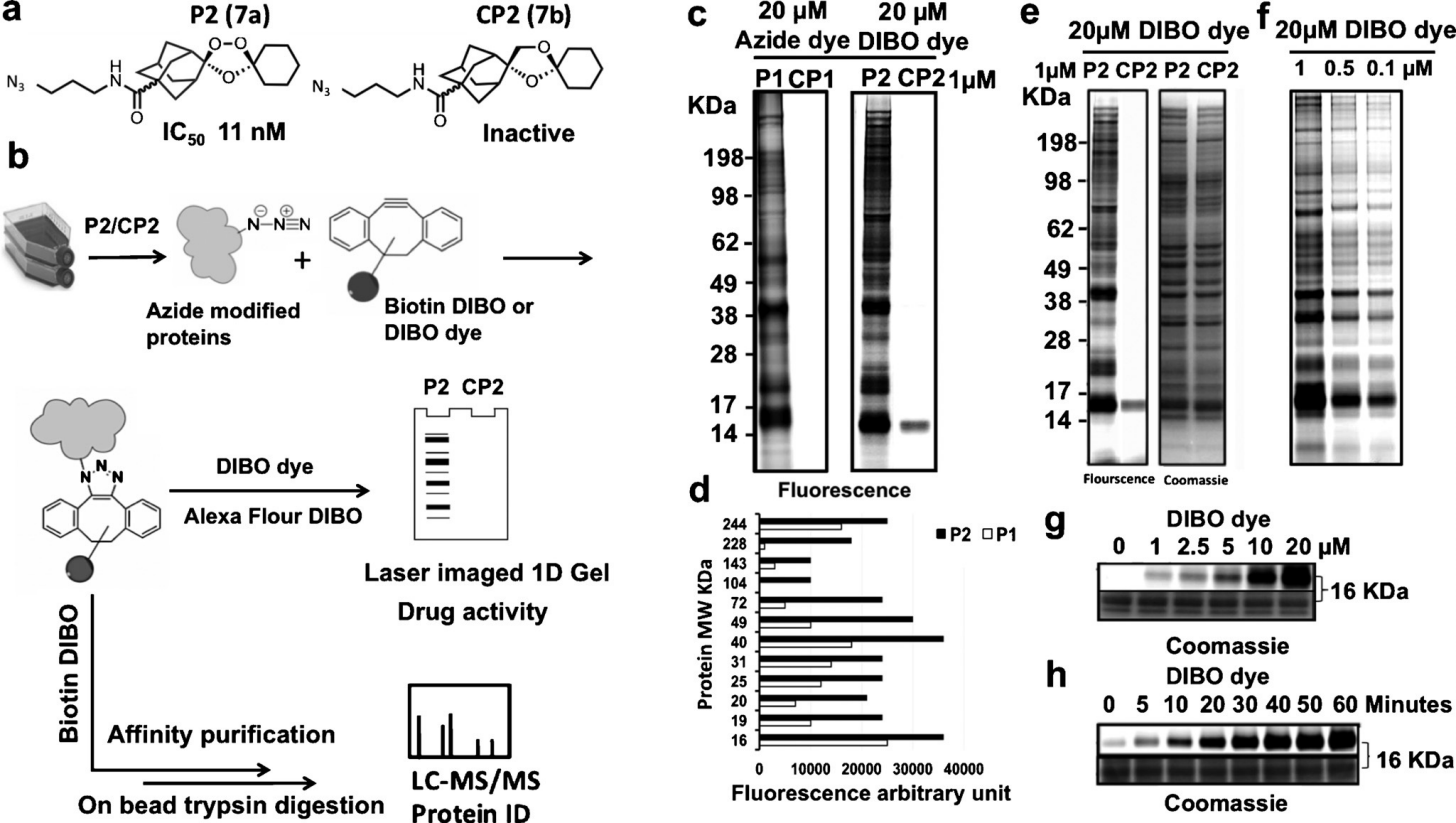
Labeling of parasite proteins *(P. falciparum*, 3D7 strain) using **7 a**. a) Chemical structure of ozonide azide probe (**P2** (**7 a**)) and deoxyether analogue (**CP2** (**7 b**)) and their antimalarial activity against *P. falciparum* 3D7. b) General workflows of copper‐free click methodology for in situ parasite protein identification using azide trioxolanes probes as detailed mentioned in methodology section. c) Fluorescence image of 1D gel for proteins labeled in situ with alkyne probes (**P1** (**6 a**) and **CP1** (**6 b**)) vs. azide probes (**P2** (**7 a**) and **CP2** (**7 b**)), note that no labeling occurs with negative control alkyne (**CP1** (**6 b**)) and azide control (**CP2** (**7 b**)). d) Arbitrary fluorescence intensity measurements of the major protein bands labeled and identified with 20 μm Alexa flour 488 azide for parasite proteins tagged with 1 μm of alkyne probe (**P1** (**6 a**)) vs. proteins tagged with 1 μm of azide probe (**P2** (**7 a**)) identified with 20 μm Click‐IT Alexa Fluor 488 DIBO Alkyne. Fluorescence arbitrary units reveal higher sensitivity in case of bio‐orthogonal copper free click reaction, that is, **P2** (**7 a**) treatment. e) Gel image of **P2** (**7 a**) treatment vs. control, pre and post coomassie stain with equal protein loading. f) Fluorescence image representing probe titration from 1 to 0.1 μm **P2** (**7 a**) probe; proteins identified via copper free click reaction with Click‐IT Alexa Fluor 488 DIBO Alkyne. No changes were observed in labeling profiles of the trioxolane‐tagged proteins with concentrations relevant to pharmacological concentration of the drug (100 nm). g) Titration of DIBO dye at various concentrations up to 20 μm for parasite treated in situ with 1 μm **P2** (**7 a**). h) Time dependent increase of fluorescence signal for proteins tagged with 1 μm **P2** (**7 a**) and 20 μm Click‐IT Alexa Fluor 488 DIBO Alkyne indicating that the maximum band intensities could be achieved after 1‐hour of click reaction incubation.

After validating the importance of the endoperoxide bridge for protein alkylation using a 1D gel, we excluded the gel electrophoresis step and advanced the method to direct analysis of the alkylated protein matrix captured using an “on‐bead” trypsin digestion protocol as shown in Figure 3 b and Figure S3. Overall, multiple proteins critical to parasite life were identified as trioxolane targets with the **P2** (**7 a**) probe (Figure S3). No labeling was evident with **CP2** (**7 b**) the negative control analogue or with the DMSO control (Figure S3). Having completed the analysis of the 1,2,4‐trioxolane proteome we carried out a head‐to‐head comparison of an analogous ART activity based profiling probe (Figure 4).^9^

**Figure 4:**
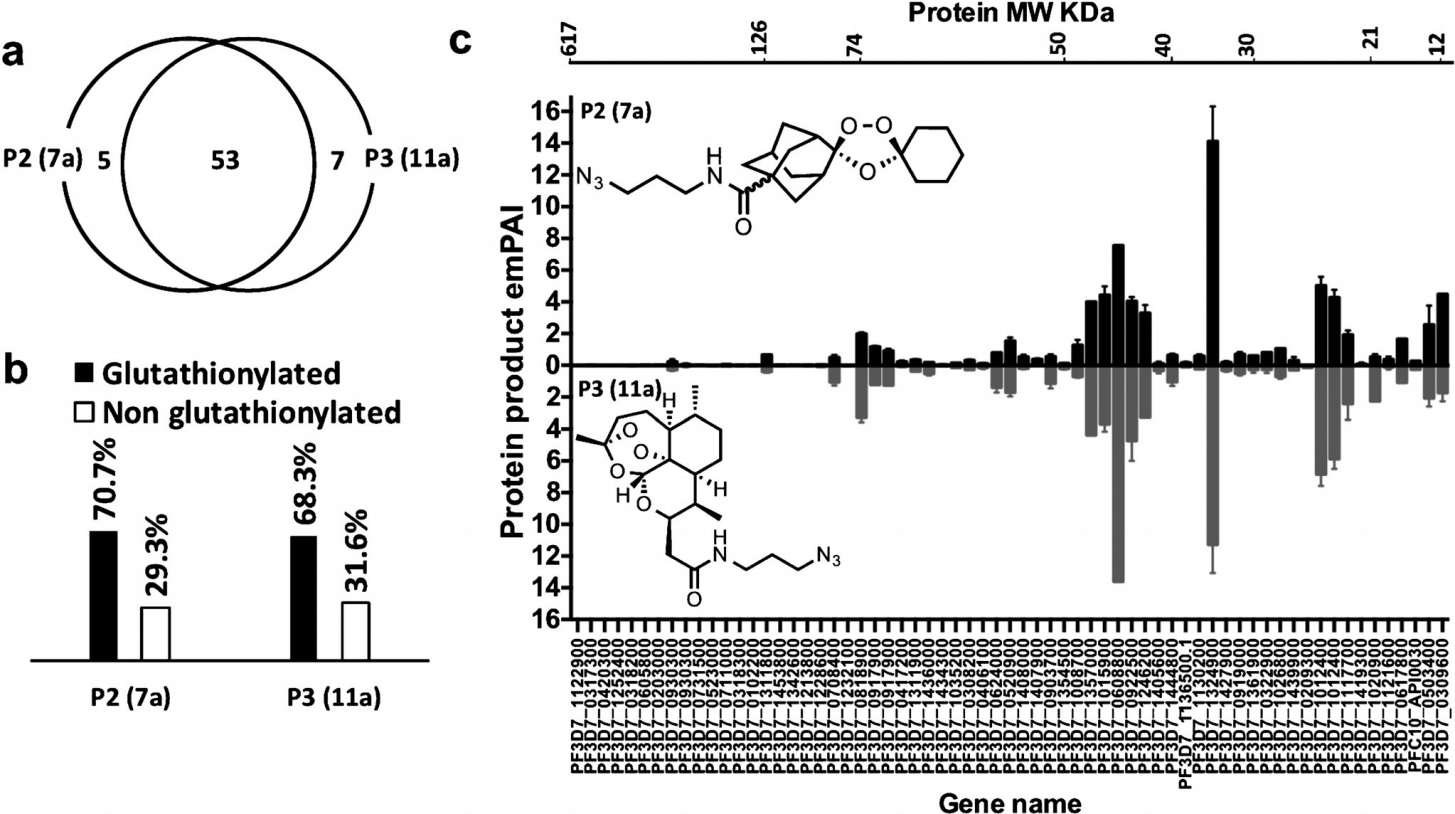
Mass spectrometry experiments with azide trioxolane azide probe (**P2** (**7 a**)) vs. artemsinin azide probe (**P3** (**11 a**)). a) Venn diagram demonstrating overlap between proteins identified with the endoperoxide probes, **P2** (**7 a**) and **P3** (**11 a**) respectively. (b) Percentage of the glutathionylated proteins, which contains the GSH binding motif that was identified with endoperoxides probes **P2** (**7 a**) and **P3** (**11 a**) in light of Kehr et al.^11^ (c) Head to head comparison between proteins identified with **P2** (**7 a**) vs. **P3** (**11 a**). Proteins sorted according to their molecular weight from high to low. Errors bars represented the standard deviation for protein quantity in each treatment calculated by dividing the exponentially modified protein abundance index (*em*PAI)^16^ for each protein by the total *em*PAI values (each treatment contain two replicate, for accuracy each replicate is the average of four injections into the Orbitrap LC‐MS/MS instrument).

Remarkably, as depicted in Figure 4, both the trioxolanes probe **P2** (**7 a**) and ART probe **P3** (**11 a**) share strongly overlapping protein‐labeling profiles both qualitatively and semi‐quantitatively. From a total of 62 proteins confidently identified with the two probes 53 of the proteins were tagged with both **P2** (**7 a**) and **P3** (**11 a**) (Figure 4 a), with no labeling observed for control probes **CP2** (**7 b**) and **CP3** (**11 b**) (Figure S3). From a mechanistic perspective it is important to note that ca. 70 % of the tagged proteins can be readily glutathionylated (Figure 4 b), a post translational modification that can effect redox regulation and signal transduction.^11^ For instance, EXP1, the membrane glutathione S‐transferase identified with both **P2** (**7 a**) and **P3** (**11 a**) was reported to efficiently degrade cytotoxic hematin in malaria parasite.^12^ Interestingly, artesunate, a water‐soluble derivative of ART, has been reported to inhibit the GST activity of EXP1 in a hematin dependent manner at ca. 2 nm (IC_50_).^12^ *Pf*EXP1 facilitates the conjugation of GSH with artesunate in vitro.^12^ We proposed that carbon centered radicals generated from the reductive scission of the endoperoxide bridge from either **P2** (**7 a**) or **P3** (**11 a**) alkylate proteins by C‐radical attack at the disulfide bridges of the glutathionylated proteins identified in this study;^11^, ^13^ further work is on‐going to confirm this mode of reactivity. The major proteins in the mass range 30 to 75 KDa identified for **P2** (**7 a**) are, *Pf*LDH, *Pf*OAT and *Pf*HGPRT, similar to that seen earlier with the equivalent ART probes,^7^, ^9^ Collectively the data suggest that 1,2,4‐trixolanes efficiently target plasmodial energy supply, the antioxidant defence system and DNA synthesis. In addition, ART has been shown to modulate a variety of signaling pathways in cancer cells.^14^ In the present study many cytoskeletal proteins including α‐tubulin, β‐tubulin, and actin 1 were labeled with trioxolanes and ART probes suggesting a potential link between these endoperoxide drugs and parasite cell structure, protein trafficking systems and signal transduction.^9^ Moreover, the global analysis of protein alkylation generated through **P2** (**7 a**), for both classes, that is, ART and trioxolanes, is consistent with the “cluster bomb” hypothesis,^7^, ^9^, ^15^ whereby Fe^2+^/heme‐activated drug alkylates multiple redox‐susceptible protein targets functioning in multiple cellular pathways (Figure S4) including the food vacuole, a site considered important for iron dependent activation, and also in the cytosol (Figure S5).

To conclude, a chemical proteomic approach has for the first time enabled formal identification of the key proteins that are alkylated by the 1,2,4‐trixolane class of antimalarial. Significantly, the proteomic profile of 1,2,4‐trioxolanes is similar to the artemisinins suggesting that 1,2,4‐trioxolanes are multi‐targeting like artemisinin and it remains to be seen if a similar stress response and accumulation of ubiquinated proteins occurs for this class of antimalarial in *Pf*Kelch13 resistant parasites.^17^

Clearly, our data raises concerns of the potential cross‐resistance^18^ between these two different antimalarial chemotypes. Our optimised endoperoxide‐ABPPs strategy has generated a specific and robust set of tools to study potential protein targets of the endoperoxide class of antimalarials.^19^ We are currently further refining this approach to accommodate a broader range of peroxide‐based antimalarial chemotypes. Work is also underway to establish the life‐cycle‐dependent “endoperoxome” patterns in asexual and sexual stages of *P. falciparum* parasite isolates with well‐characterized artemisinin drug resistance phenotypes to assist in our understanding of this worrying clinical phenomenon.

## Supporting information

As a service to our authors and readers, this journal provides supporting information supplied by the authors. Such materials are peer reviewed and may be re‐organized for online delivery, but are not copy‐edited or typeset. Technical support issues arising from supporting information (other than missing files) should be addressed to the authors.

Supplementary
