# Supplementary material for: A Click Chemistry‐Based Proteomic Approach Reveals that 1,2,4‐Trioxolane and Artemisinin Antimalarials Share a Common Protein Alkylation Profile
Source: Angew Chem Int Ed Engl. 2016 Apr 18;55(22):6401–5. doi: 10.1002/anie.201512062 (PMC4934138; doi:10.1002/anie.201512062)
Supplement: Supplementary file 1 — Supplementary [file ANIE-55-6401-s001.pdf]

Angewandte Chemie
Eine Zeitschrift der Gesellschaft Deutscher Chemiker

Supporting Information

A Click Chemistry-Based Proteomic Approach Reveals that 1,2,4-Trioxolane and Artemisinin Antimalarials Share a Common Protein Alkylation Profile

*Hanafy M. Ismail, Victoria E. Barton, Matthew Panchana, Sitthivut Charoensutthivarakul, Giancarlo A. Biagini, Stephen A. Ward, and Paul M. O'Neill**

anie_201512062_sm_miscellaneous_information.pdf

SUPPORTING INFORMATION

Synthesis and Analysis of click probes

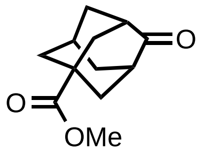

3

5-Carbomethoxyadamantan-2-one 3. A solution of oleum (30 % SO_3, 48 mL, 100 mmol) at 60 ºC was added (drop-wise) to 5-hydoxy-2-adamantanone (2.0 g, 12 mmol) in formic acid (99 %, 12 mL, 0.32 mmol) and the solution stirred vigorously for 40 minutes. After this time additional formic acid was added (99 %, 12 mL, 0.32 mmol) over 40 minutes and the solution was allowed to stir for a further 60 minutes. The residue was then cautiously poured into vigorously stirred methanol (100 mL) at 0 °C. After 2 hours at room temperature the residue was concentrated and poured over ice (~5 g). The aqueous layer was washed with CH_2Cl_2 (3 x 30 mL) and combined organic fractions washed with brine (2 x 50 mL), dried ($MgSO_4$) and concentrated *in vacuo*. The crude product was purified by column chromatography (40:60 EtOAc/*n*-Hex) to yield (1.6 g (65 %)) as a white crystalline solid; m.p 104-105 °C (50-51°C (solv:hexane [1])); ^{1}H NMR (400 MHz, $CDCl_3$) δ 3.68 (3H, s, OCH_3) 2.59 (2H, s, 2 x C-H, bridgehead), 2.19-1.97 (11H, m); ^{13}C NMR (100 MHz, $CDCl_3$) *δ* 216.8 (C=O), 176.5 ($\underline{C}O_2CH_3$), 52.3 ($O\underline{C}H_3$), 46.1, 40.7, 40.5, 38.7, 38.2, 27.7; IR/(nujol)cm^{-1}; 2859, 2829, 1716 (C=O) ; HRMS (CI) 226.14482 $[M+NH_4]^+$ $C_{12}H_{20}O_3N$ requires 226.14430.

Cyclohexanone (*O*-methyl) oxime 4 To a solution of cyclohexanone (2.1 mL, 20 mmol) in methanol (20 mL) was added anhydrous pyridine (2.4 mL, 30 mmol) and *O*-methylhydroxylamine hydrochloride (2.5 g, 30 mmol). The reaction mixture was allowed to stir under nitrogen for 24 hours, diluted with CH_2Cl_2 (50 mL) and water (50 mL). The organic layer was separated and the aqueous layer extracted with CH_2Cl_2 (2 x 50mL). The combined organic extracts were washed with 1M HCl (2 x 50 mL) and saturated NaCl (30 mL) and dried over $MgSO_4$. The majority of the solvent was removed by distillation at 40 °C. The remaining traces of CH_2Cl_2 were removed by Kugelrohr distillation to give **4** (2.1 g (82 %)) as colourless oil.

4: ^{1}H NMR (400 MHz, $CDCl_3$) δ 3.82 (3H, s , CH_3), 2.46-2.44 (2H, t, *J*=6.2 Hz, CH_2), 2.21 (2H, t, *J*=6.4 Hz, CH_2), 1.68-1.54 (6H, m); ^{13}C NMR (100 MHz, $CDCl_3$); δ 160.5 (C=N), 61.3 (OCH_3), 32.5 (CH_2), 27.4 (CH_2), 26.2, 26.1, 25.5; IR (neat)/cm^{-1} 1641 (C=N) ; HRMS (CI) 128.10787 $[M+H]^+$ $C_7H_{14}NO$ requires 128.10754.

5-Adamantane-2-spiro-1’ 2’ 4’-trioxaspiro [4.5] decane methyl ester 5

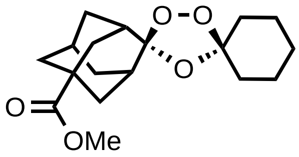

5

Ozone (3-4 %) was produced with an ozone generator, first passed through CH_2Cl_2 (80 mL), cooled to -78°C, then bubbled through a solution of **4** (0.50 g, 3.96 mmol) and **3** (0.55 g, 2.6 mmol) in pentane (90 mL). The reaction was left for 2.5 hours. After completion the solution was flushed with nitrogen for 15 min then concentrated *in vacuo.* The residue was purified by column chromatography (1:99 EtOAc/*n*-Hex) to give **5** (0.26 g, 30 %) as colourless oil.

5: ^{1}H NMR (400 MHz, $CDCl_3$) δ 3.65/3.63 (ratio 1:1) (3H, s, OCH_3), 2.23-1.23 (23H, m); ^{13}C NMR (100 MHz, $CDCl_3$) δ 177.6/177.5 (C=O), 110.5 (<u>C</u>-O-O-C), 109.6(<u>C</u>-O-O-C), 52.0 (OCH_3), 40.3, 39.9, 38.6, 36.7, 36.5, 36.4, 36.3, 35.1, 34.1, 34.0, 30.2, 27.0, 26.6, 25.7, 25.3, 24.2, 22.8; IR (neat) /cm^{-1} 2935, 2861, 1729 (C=O), 1450, 1326, 1241, 1187, 1112, 1095, 939(O-O); HRMS (ESI) 345.1685 $[M+Na]^+$; $C_{18}H_{26}O_5{}^{23}Na$ requires 345.1678.

5-Adamantane carboxy-2-spiro--1’-2’-4’-trioxaspiro[4.5]decane

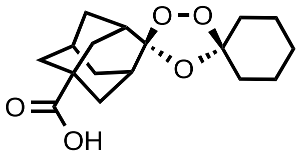

To a solution of **5** (0.63 g, 1.8 mmol) in 95 % ethanol (10 mL) was added 15 % aq. NaOH (10 mL). The mixture was allowed to stir for 3 hours and acidified with 3 M HCl (10 mL). Water (10 mL) and EtOAc (20 mL) was added to the solution and the aqueous layer was washed with EtOAc (3 x 20 mL). The combined organic extracts were washed with water (30 mL), sat. aq. $NaHCO_3$ (20 mL) and brine (20 mL), dried ($MgSO_4$), filtered and concentrated to give compound 67 (200 mg, 83 %) as fine white powder.

m.p 132-135 °C; ^{1}H NMR (400 MHz, $CDCl_3$) δ 2.25-2.22 (1H, m, CH), 2.15-2.12 (1H, m, CH), 2.06-1.81 (7H, m), 1.33-1.19 (1H, m), 1.74-1.32 (13H, m) ; ^{13}C NMR (100 MHz, $CDCl_3$) *δ* 183.6 (<u>C</u>OOH), 110.3 (O-C-O), 109.8 (O-C-O), 40.0 (H_2<u>C</u>-COOH), 39.6, 38.3, 38.2, 36.4, 36.3, 36.2, 36.1, 35.1, 34.0, 26.8, 26.4, 25.3, 24.3, 24.2 ; IR (neat)/cm^{-1} 3223 (OH), 2935, 1693 (C=O) 1523, 1450, 1257, 1116, 1079, 941, 742 (O-O), 611 (O-O) ; HRMS (ESI) 307.1530 $[M-H]^-$ $C_{17}H_{23}O_5$ requires 307.1545 ; $C_{17}H_{24}O_5$ requires C 66.21 % H 7.84 found C 66.01% H 8.01%.

General Coupling Procedure 1: To a stirring solution of carboxylic acid (50 mg, 0.15 mmol) in CH_2Cl_2 (10 mL) was added EDC.HCl (50 mg, 0.24 mmol), and DMAP (19 mg, 0.17 mmol). After activating for 1 hour, either amine azide or amine alkyne (38 mg, 19 mmol) in CH_2Cl_2 was added to the solution. After 24 hours the solvent was removed under reduced pressure and the residue purified by flash column chromatography (silica gel, 20:80 EtOAc/*n*-Hex) gave desired product as colourless crystals.

Alkyne Probe P1(6a): Synthesis achieved using General Procedure 1 to give title compound (44 mg, 80 %) as colourless crystals.

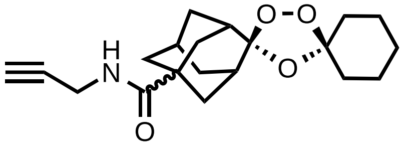

P1(6a): Rf =0.54 (40% EtOAc/hexane); ^{1}H NMR (400 MHz, $CDCl_3$) δ 5.73 (1H, br s, NH) 4.05-4.01 (2H, m, CH_2), 2.25-1.36 (26 H, m); ^{13}C NMR (100 MHz, $CDCl_3$) *δ* 178.2 (<u>C</u>ONH), 155.4, 110.5 (O-C-O), 109.6 (O-C-O), 56.2, 50.2, 44.3, 39.3, 36.6, 35.3, 35.1,

34.0, 33.0, 31.7, 27.0, 26.6, 25.8, 25.3, 25.1, 24.2 ; $C_{20}H_{27}NO_4$ requires C 69.54 % H 7.88 % N 4.05 % found C 69.31 % H 8.05 % N 3.62 %.

Synthesis of 3-azido-1-propylamine To a stirred solution of 3-chloropropylamine hydrochloride (2.2 g, 17mmol) in water (20 mL) was added sodium azide (3.4 g, 48 mmol) and the solution was heated to 80°C. After 15 hours the solution was cooled and basified (KOH). EtOAc (20mL) was added and the organic layer was extracted (3 x 15 mL). The organic layer was dried over $MgSO_4$ and solvent removed *in vacuo.* The product was contaminated with starting material therefore flash column chromatograpy (20-40% EtOAc/Hex) was necessary to obtain the pure product as a colourless oil (0.5 g, 29 %).

^1H NMR (400 MHz, $CDCl_3$) δ 3.40 (2H, t, J= 6.6 Hz, $\underline{CH_2}N_3$), 2.87-2.83 (2H, m, $\underline{CH_2}NH_2$), 1.86 (2H, br s, $\underline{NH_2}$), 1.81-1.74 (2H, m, CH_2); ^{13}C NMR (100 MHz, $CDCl_3$) 49.4 (C-N_3), 39.5 (C-NH_2), 32.0 (CH_2); HRMS (CI) 101.1, $[M+H]^+$ requires 101.1.

Azide Probe P2(7a): Synthesis achieved using general procedure 1 to give title compound (44 mg, 70 %) as colourless crystals.

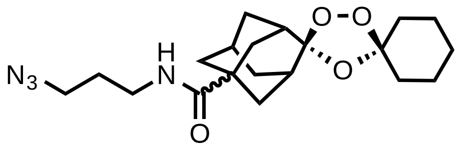

P2(7a): Rf = 0.38 (40% EtOac/hexane) ^1H NMR (400 MHz, $CDCl_3$) δ 5.84 (1H, br s, NH) 4.39-4.30 (4H, m, HN-CH_2 and H_2C-N_3), 2.08-1.36 (26 H, m) ; ^{13}C NMR (100 MHz, $CDCl_3$) δ 177.3 ($\underline{C}$ONH), 110.4 (O-C-O), 109.8 (O-C-O), 50.0, 49.9, 40.2, 39.7, 38.8, 37.6, 37.5, 37.0, 36.5, 35.1, 34.1, 34.0, 29.0, 27.1, 26.7, 25.3, 24.2 ; HRMS (ESI) 413.2163 $[M+Na]^+$ $C_{20}H_{30}N_4O_4$ ^{23}Na requires 413.2165 ; $C_{20}H_{30}N_4O_4$ requires C 61.52 % H 7.74 % N 14.35 % found C 61.83 % H 7.85 % N 14.39 %.

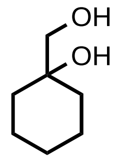

9

9: To a solution of methylenecyclohexanone (1g, 10mmol) in Acetone/DCM (1:1, 20mL) was added OsO4 (0.12g, 0.5mmol) and NMO (1.5g, 13 mmol) and the solution was left to stir for 24 h at room temperature. The solvent was removed and crude material purified by flash column chromatography (20-50% EtOAc/Hexane) to give 550 mg (43 %) colourless crystals.

9: ^{1}H NMR (400 MHz, $CDCl_3$) *δ* 3.45 (2H, s, CH_2), 2.29 (2H, br s, OH), 1.66-1.26 (10 H, m, cyclohex CH); ^{13}C NMR (100 MHz, $CDCl_3$) δ 72.2 (C-OH), 70.5 (C-OH), 34.5, 26.2, 22.2. $C_7H_{14}O_2$ requires 148.3 $[M+H]^+$; found 148.3.

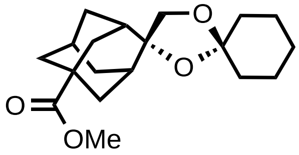

10

10: To a solution of Adamantanone methyl ester (416mg, 2mmol) in DCM (10 mL) was added **9** (200 mg, 1.5 mmol) and PTSA (400 mg, 2.1 mmol) and stirred for 8 hours. After **9** had been consumed the solvent was removed and crude material purified by flash column chromatography (20-50% EtOAc/Hexane) to give 250 mg (52 %) as colourless oil.

10: ^{1}H NMR (400 MHz, $CDCl_3$) *δ* 3.73 (1H, s, CH_3), 3.65 (2H, s, CH_2), 2.24-2.26 (25 H); ^{13}C NMR (100 MHz, $CDCl_3$) δ 178.1 (CONH), 110.6, 80.9, 60.7, 51.9, 40.3, 39.9, 38.9, 38.2, 38.0, 37.9, 37.5, 37.3, 36.7, 34.2, 33.9, 27.1, 26.9, 25.8, 24.0. $C_{19}H_{28}O_4{}^{23}Na$ requires 343.1885 $[M+Na]^+$ 343.1895.

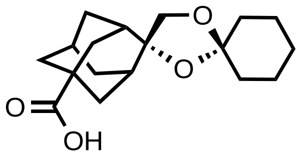

Acid Intermediate: To a solution of **10** (150 mg, mmol) in EtOH (95%) was added NaOH (15%) and stirred for 3h. The solution was acidified with 1.0 M aq. HCl and ethanol was removed and water (10mL) added to the residue. The aqueous layer was extracted with CH_2Cl_2 (3 x 30 mL). The combined organic extracts were dried over $MgSO_4$, filtered and concentrated under reduced pressure to give the acid (100 mg, 92 %) as a colourless brittle solid.

Acid Intermediate: ^{1}H NMR (400 MHz, $CDCl_3$) *δ* 3.73 (2H, s, CH_2), 2.27-2.18 (2H, m), 2.01-1.26 (21 H); ^{13}C NMR (100 MHz, $CDCl_3$) δ 178.1 (COOH), 110.6, 80.9, 60.7, 51.9, 40.3, 39.9, 38.9, 38.2, 38.0, 37.9, 37.5, 37.3, 36.7, 34.2, 33.9, 27.1, 26.9, 25.8, 24.0; MS (EI) 329.1727 $[M+Na]^+$ $C_{18}H_{26}O_4$ ^{23}Na requires 329.1729.

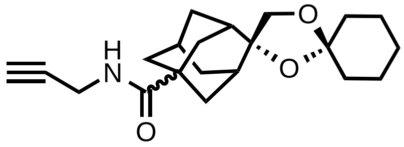

CP1(6b): Synthesis achieved using General Procedure 1 to give title compound (77 mg, 70 %) as a colourless oil.

CP1 (6b): ^{1}H NMR (400 MHz, $CDCl_3$) δ 5.79 (1H, br s, NH) 4.03-4.01 (2H, m, CH_2), 3.74 (2H, s, CH_2), 2.25-1.24 (24 H, m) ; ^{13}C NMR (100 MHz, $CDCl_3$) *δ* 177.3 (<u>C</u>ONH), 110.5 (O-C-O), 81.1 (O-CH_2), 73.8, 71.9, 60.7, 40.2, 39.8, 38.8, 37.3, 36.9, 34.0, 29.6, 27.1, 26.8, 25.8, 24.1; HRMS (ESI) 366.2048 $[M+Na]^+$ $C_{21}H_{29}NO_3$ ^{23}Na requires 366.2045; $C_{21}H_{29}NO_3$ requires C 73.44 % H 8.51 % N 4.08 % found C 73.33 % H 8.49 % N 4.13 %.

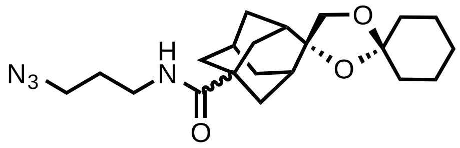

CP2 (7b): Synthesis achieved using general procedure 1 to give title compound (60 mg, 48 %) as colourless oil.

CP2 (7b): ^{1}H NMR (400 MHz, $CDCl_3$) δ 5.86 (1H, br s, NH) 3.74 (2H, s, O-CH_2), 3.38-3.29 (4H, m, HN-CH_2 and CH_2-N_3), 2.17-1.35 (25 H, m); ^{13}C NMR (100 MHz, $CDCl_3$) *δ* 178.0 (<u>C</u>ONH), 110.6 (O-C-O), 81.0 (C-O), 73.8, 50.0 (C-N_3), 49.8, 40.2, 39.9, 38.3, 37.5, 34.1, 29.1, 27.2, 26.9, 25.7, 25.8, 24.1, 21.4; HRMS (ESI) 411.2367 $[M+Na]^+$ $C_{21}H_{32}N_4O_3$ Na requires 411.2372 ; $C_{21}H_{32}N_4O_3$ requires C 64.63 % H 8.30 % N 14.42 % found C 64.63 % H 8.30 % N 14.39 %.

Parasite culture *Plasmodium falciparum* (3D7 strain) parasites were cultured following the method of Trager and Jensen [2] with modifications. The cultures consisted of a 4% (v/v) suspension of O+ erythrocytes in RPMI 1640 medium (R8758, glutamine and $NaHCO_3$) supplemented with 10% pooled human AB+ serum, 25 mM HEPES (pH 7.4) and 20 µM gentamicin sulphate. Parasite cultures were maintained at 37°C in 3% CO_2, 4% O_2, and 93% N_2 atmosphere. Parasite growth was synchronized by treatment with sorbitol[3]. The culture was evaluated for parasitemia and parasite stages daily using a Giemsa staining microscopy method.

Drug sensitivity assay The antimalarial activities of standards and probes were evaluated using standard fluorometric DNA binding method[4]. Assay plates were created in 96-well plates by Hamilton Star robotic platform, concentration ranged from 2 µM to 0.122 nM with 1 µM artemisinin as a positive control. Parasite inoculum was 0.5 % parasitemia (ring stage) at 1% haematocrit and compound concentration range from 1 µM to 0.61 nM. Parasites were incubated with compounds for 48 hours in culture condition. Parasite growth were assessed by measurement of the intensity of SYBR

Green I fluorescent DNA staining dye at 490/520 nm excitation/emission wavelength. The half maximal inhibitory concentration (IC_{50}) was calculated from the log of the dose– response relationship, as fitted with Grafit software (Erithacus Software, Horley, UK). Probes, respective controls and Artemisinin stock solutions (5 mM) were prepared in dimethyl sulphoxide (DMSO). The results are given as the means of at least three separate experiments.

Parasite treatment for chemical proteomic analysis Ten flasks of synchronized trophozoites stage parasites cultures at 10-12% parasitemia, 4% hematocrit (0.5 L of culture) were treated with 100 µl of 5 mM endoperoxide probes (**P1 (6a), P2 (7a), P3 (11a)** and their respective controls (**CP1 (6b), CP2 (7b) and CP3 (11b)**) to give a final dilution of 1 µM probe per treatment. All treatments were carried out with a minimum of two replicates, unless noted otherwise. Parasite cultures were maintained at 37°C in 3% CO_2, 4% O_2, and 93% N_2 atmosphere for 6 hours. An equivalent culture without drug (control) in five replicates was treated with 10 µl of dimethyl sulfoxide (DMSO) as a control treatment maintained under identical culture conditions. Parasite proteins were sequentially extracted using a modification of the protocol described by Molley *et al.*[5]. Serum proteins were removed by three washes with sterile Ca^{2+} and Mg^{2+} free Dulbecco's Phosphate Buffered Saline pH 7.4 (D-PBS; Invitrogen) containing sorbitol (250 mM sorbitol) followed by centrifugation at 2000x*g* at 4°C for 5 min. The parasites were released from infected erythrocytes by saponin lysis (0.15% w/v in D-PBS-buffered sorbitol) for 10 minutes on ice and then washed three times with ice-cold D-PBS-buffered sorbitol, followed by centrifugation at 5000x*g* for 15 min to sediment parasites from red cell membrane debris. Parasite pellets were stored at -80°C until protein extraction. In all experiments, proteins were extracted by subjecting the parasite to three cycles of freeze-thaw in D-PBS pH 7.4 containing (0.1 mM DTT, 1% Nonidet P-40 (NP-40; Sigma-Aldrich, UK), and protease inhibitor cocktail (*c*Omplete EDTA-free Protease Inhibitor Cocktail; Roche, Mannheim, Germany)). This was followed by trituration with a sonicator probe on ice (~4°C). The PBS-soluble fraction was then separated (10000x*g* at 4°C for 15 min) and the supernatant stored at -80°C for later use. Protein concentration was determined by the method of Bradford [6].

In gel fluorescence analysis of endoperoxides tagged protein(s) from *P. falciparum* 3D7.

Copper based click reaction In all experiments *P. falciparum* protein extracts were adjusted to 2 mg/ml by diluting the protein extract in DPBS. Click reactions were performed by treatment of 44 μl of the samples twith alkyne endoperoxide probes and respective controls (deoxyether probe or DMSO solvent) in duplicates with 0.5 μL Alexa Fluor 488 azide reporter (2 mM stock solution in DMSO) to give 20μM reporter per reaction (total volume 50 μl). For optimum concentration of the reporter, Alexa Fluor 488 azide titration was examined at various concentrations of the reporter giving final dilutions of 10, 20, 50 and 100μM per reaction. In all cases, after addition of the reporter, the click chemistry reaction proceeded with conditions optimized by Speers and Cravatt.[7] Briefly, all samples were treated with Alexa Fluor 488 azide reporter, followed by 1 μL freshly prepared Tris(2-carboxyethyl) phosphine hydrochloride (50 mM stock in water) and 3.3 μL ligand (1.7 mM stock in DMSO:*t*-butanol 1:4), giving a *t*-butanol concentration of 5% (vol/vol). Samples were gently vortexed, and 1 μL CuSO4 (50 mM stock in water) was added. Each reaction was then gently vortexed and allowed to react at room temperature for 1 h in the dark, with regular vortexing every 15 min. NuPAGE Novex 4–12% Bis-Tris Gels (Invitrogen) were used for protein separation, whereby 2X SDS/PAGE loading buffer (reducing) was added to each reaction, the samples were heated at 90 °C for 8 min, and then 15 μL per well were loaded onto gel. The Gel was mainitained (5:4:1 water/methanol/acetic acid) overnight in the dark, and fluorescence intensities were measured using an Ettan DIGE Laser imager (GE Healthcare) with a CY2 filter. Equal protein loading was confirmed by post scan staining with Gel code Blue Coomassie stain (Pierce, UK).

Copper free click reaction for the samples treated with azide probes (**P2 (7a)** and **P3 (11a)**) and controls including DMSO and deoxyether azide controls (**CP2 (7b)** and **CP3 (11b)**), Click-IT® Alexa Fluor 488 DIBO Alkyne (Invitrogen, UK) was used as a reporter at final dilution 20μM in final volume 50 μl reaction. Copper-free click reaction conditions were processed without the addition of TCEP, TBTA and CuSO4[8]. An adequate mix was applied using vortex mixer after adding Click-IT® Alexa Fluor 488 DIBO Alkyne.

Then the reaction was incubated for 1 hour at room temperature in the dark with gentle mixing every 15 min. After 1 hour, the click reaction was terminated by addition of 2X SDS/PAGE loading buffer (reducing) to each reaction, the samples were heated at 90°C for 8 min, Alexa flour 488 tagged proteins from treatments and control were separated and visualized as mentioned earlier.

For identifying the optimum concentration of the reporter, titration of the Click-IT® Alexa Fluor DIBO Alkyne concentration was carried out by considering various concentrations giving final dilutions of 1, 2.5, 5,10 and 20µM per reaction.

Time point titration of the click reaction was carried out by running the reaction with **P2 (7a)** tagged proteins at 2 mg/ml with 20µM of Click-IT® Alexa Fluor DIBO Alkyne in total volume of the 500µl. At various time points; 0, 5, 10, 20 30, 40, 50 and 60 minutes the reaction terminated by transferring 50µl of the solution and mixed with 2X loading buffer. Samples heated and 1D-Gel analysis conceived as mentioned above.

Identification of endoperoxide tagged protein(s) from *P. falciparum* 3D7 using LC-MS/MS analysis In all cases *P. falciparum* protein extracts were adjusted to 2 mg/ml by diluting the protein extract in DPBS in a 1 ml total volume. Each sample was split into two halves (500µl each) and the click reaction initiated by the addition of reporter under relevant click chemistry conditions. For the samples treated with alkyne probe, biotin-azide (Invitrogen, UK) was used as a reporter in the presence of reduced copper as a catalyst according to conditions optimized by Speers and Cravatt [7]. Briefly to each tube contained 500µL, 5.65 µl of 5 mM biotin-azide, 11.3 µL, 50 mM TCEP (tris (2-carboxyethyl) phosphine), 34 µL, 1.7 mM TBTA (Tris [(1-benzyl-1H-1, 2,3-triazol-4-yl) methyl] amine), and 11.3 µL, 50 mM CuSO4, each component was added in the order. An adequate mix was applied using a vortex mixer after adding each component with an exception for TCEP.

For samples treated with endoperoxide azide probes, biotin azides were replaced with Click-IT® Biotin DIBO Alkyne (Invitrogen, UK) to give final dilution of 20µM as mentioned above. In all cases the reaction was incubated for 1 hour at room temperature in the dark with gentle mixing every 15 min. After 1 hour, click reaction was

terminated, and excess components removed as follows. The respective sample was combined and protein precipitated with cold methanol and centrifuged at 6,500g for 4 min at 4°C. Then pellets were washed with 750-μL cold methanol and sonicated 3-4 s, three times. Then 650 μL 2.5% Sodium dodecyl sulphate (SDS; Sigma-Aldrich, UK) in D-PBS was added to the samples and sonicated 3-4s to dissolve all remaining pellet. The samples were then heated at 95°C for 5 min in a heat box and sonicated twice for 3-4 s afterward. At this step, most proteins were soluble in the solution. Samples were centrifuged, and supernatants were collected and adjusted to 3.5 mL with D-PBS. Samples stored at -20°C for further analysis**.**

Probe-labeled proteins were enriched and affinity purified, after the click reaction incubation step, using streptavidin agarose beads. An on-bead trypsin digestion protocol was performed on purified protein as described previously[9]. The tryptic peptide was subjected to high-resolution mass spectrometry peptide sequencing by using Thermo Scientific UltiMate 3000LC chromatography system coupled to Scientific LTQ Orbitrap *Velos* (Thermo Scientific, UK). To increase the accuracy of protein identification, tryptic peptides from each sample were injected into four replicates on reverse phase liquid chromatography (RPLC) according to published conditions[10]. Protein identification performed on MASCOT search engine with the following parameters: precursor mass tolerance of 7 ppm, fragment ion tolerance 0.3 Da with 2 tryptic missed cleavage permitted. Carbamidomethyl (C) set as a static modification with oxidation of methionine (M) and deamidation (N, Q) set as dynamic modifications. A decoy database searched, and relaxed peptide confidence filters applied to the dataset (ion scores $P < 0.05$ / FDR 5%). Proteins identified with minimum of one peptide were presented in Dataset 1. The exponentially modified protein abundance index (*em*PAI) was used to provide semi-quantitation of protein abundance[11]. Further bioinformatics analysis was used to identify the cellular components targeted by the trioxolanes probes using Cytoscape software [12] with CluGO plugin [13].

Table S1. In vitro antimalarial activities of Probes and Parent Compounds

Compound	*In vitro* IC50 (nM) (3D7)
**OZ277**	3.5 ± 0.2
**OZ439**	5.2 ± 0.6
**P1 (6a)**	11.5 ± 2.3
**CP1 (6b)**	NA
**P2 (7a)**	11.0 ± 4.5
**CP2 (7b)**	NA
**Artesunate**	2.3 ± 0.5

NA = not active

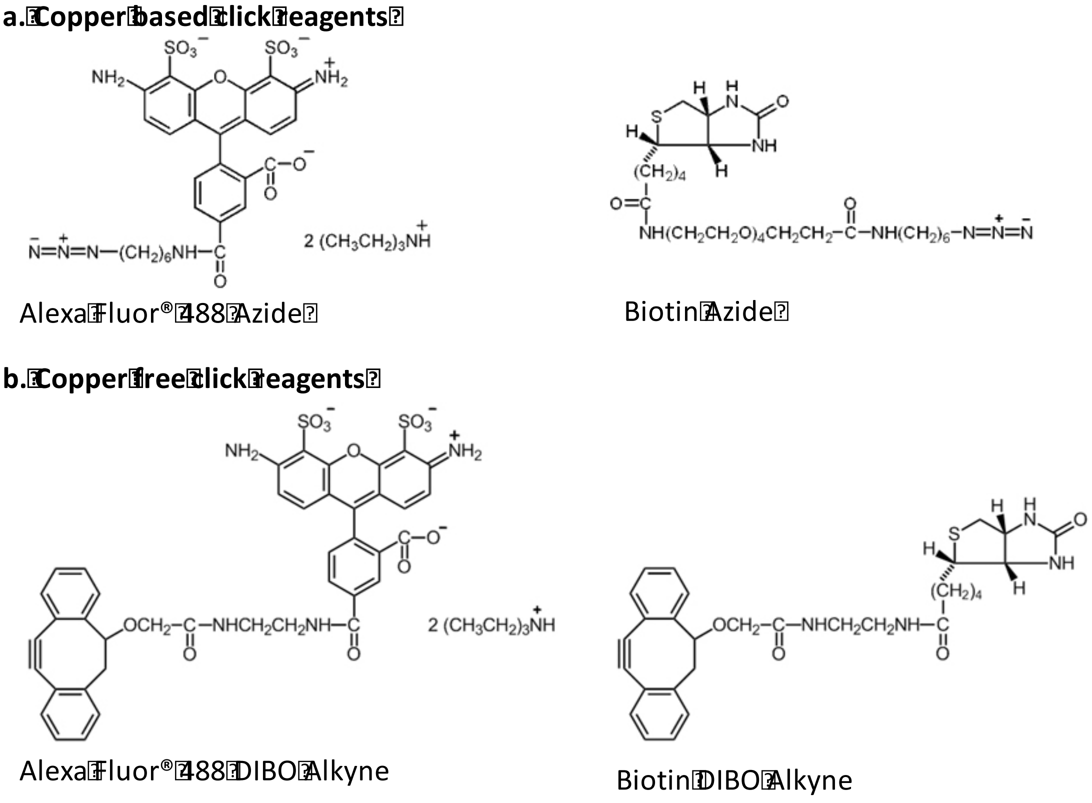

***Figure S1*. Copper based and copper free click reagents used in the study.**

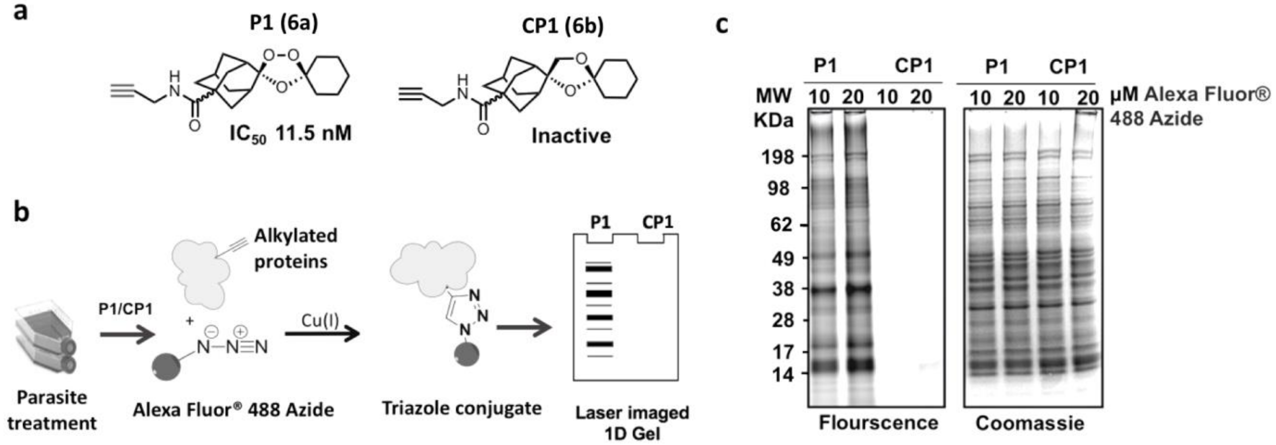

Figure S2 Click chemistry approach to identifying protein targets of the 1,2,4-trioxolane probe **P1 (6a)**.

(a) Structures and *in vitro* antimalarial activities of ozonide alkyne probe **P1 (6a)** *vs.* its deoxyether analogue **CP1 (6b)** in *P. falciparum* 3D7. (**b**) General workflow of the copper mediated click methodology for in *situ* parasite protein identification using alkyne endoperoxide probe. **(c)** Fluorescent 1D gel image of proteins labelled in *situ* with **P1 (6a)** and **CP1 (6b)**. Labelling is totally restricted to the active probe **P1 (6a)** with no control **CP1 (6b)** labelling highlighting the role of the endoperoxide function. Note equivalence of protein loading in the Coomassie gels.

MW KDa	Gene Name	Protein description	GSH binding
617.4	PF3D7_1122900	Dynein heavy chain-like protein PF11_0240	-
402.9	PF3D7_0317300	Protein PF14_0175	-
400.1	PF3D7_0420300	AP2/ERF domain-containing protein PFD0985w	-
340.5	PF3D7_1252400	Reticulocyte-binding protein 3	-
278.7	PF3D7_0318200	DNA-directed RNA polymerase II subunit RPB1	-
268.0	PF3D7_0605800	Probable DNA repair protein RAD50	-
237.8	PF3D7_0203000	Uncharacterized protein PFB0145c	-
195.7	PF3D7_0930300	Merozoite surface protein 1	+
174.6	PF3D7_0731500	Erythrocyte-binding antigen 175	-
162.3	PF3D7_0523000	Multidrug resistance protein	+
142.1	PF3D7_0711000	Putative cell division cycle ATPase	-
133.0	PF3D7_0318300	Uncharacterized protein PFC0810c	-
126.5	PF3D7_0102200	Ring-infected erythrocyte surface antigen	+
126.1	PF3D7_1311800	M1 family aminopeptidase	+
107.0	PF3D7_1453800	Bifunctional glucose-6-phosphate 1-dehydrogenase/6-phosp	-
92.3	PF3D7_1342600	Myosin-A	-
86.6	PF3D7_1213800	Proline--tRNA ligase	-
86.6	PF3D7_1228600	101 kDa malaria antigen	-
86.2	PF3D7_0708400	Heat shock 90 kDa protein homolog (Fragment)	-
81.5	PF3D7_1232100	Chaperonin CPN60, mitochondrial	+
73.9	PF3D7_0818900	Heat shock 70 kDa protein	+
72.4	PF3D7_0917900	78 kDa glucose-regulated protein homolog (Heat shock)	+
71.7	PF3D7_0417200	Bifunctional dihydrofolate reductase-thymidylate synthase	+
68.6	PF3D7_1311900	V-type proton ATPase catalytic subunit A	+
67.4	PF3D7_1436000	Glucose-6-phosphate isomerase	+
66.1	PF3D7_1434300	STI1-like protein	-
62.9	PF3D7_1035200	S-antigen protein	-
59.6	PF3D7_0308200	T-complex protein 1 subunit eta	+
55.8	PF3D7_0406100	V-type proton ATPase subunit B	-
55.3	PF3D7_0624000	Hexokinase	+
53.8	PF3D7_0520900	Adenosylhomocysteinase	+
51.5	PF3D7_1408000	Plasmepsin-2	+
51.5	PF3D7_1407900	Plasmepsin-1	+
50.3	PF3D7_0903700	Tubulin alpha chain	-
50.1	PF3D7_1354500	Adenylosuccinate synthetase	+
49.8	PF3D7_1008700	Tubulin beta chain	-
49.0	PF3D7_1357000	Elongation factor 1-alpha	+
48.7	PF3D7_1015900	Enolase	-
46.1	PF3D7_0608800	Ornithine aminotransferase	+
45.4	PF3D7_0922500	Phosphoglycerate kinase	+
41.9	PF3D7_1246200	Actin-1	+
40.6	PF3D7_1405600	Ribonucleoside-diphosphate reductase small chain	+
40.1	PF3D7_1444800	Fructose-bisphosphate aldolase	-
37.6	PF3D7_1136500.	Casein kinase I	+
35.0	PF3D7_1130200	60S acidic ribosomal protein (Ribosomal phosphoprotein P0	+
34.1	PF3D7_1324900	L-lactate dehydrogenase	+
33.0	PF3D7_1427900	Acidic leucine-rich nuclear phosphoprotein 32-related protei	-
31.8	PF3D7_0919000	Aspartic acid-rich protein	+
30.6	PF3D7_1361900	Proliferating cell nuclear antigen	+
30.0	PF3D7_0322900	40S ribosomal protein S3a	+
29.9	PF3D7_1026800	40S ribosomal protein SA	+
27.9	PF3D7_1439900	Triosephosphate isomerase	+
27.2	PF3D7_0209300	2-C-methyl-D-erythritol 2,4-cyclodiphosphate synthase	-
26.4	PF3D7_1012400	Hypoxanthine-guanine-xanthine phosphoribosyltransferase	+
24.9	PF3D7_1117700	GTP-binding nuclear protein Ran	+
24.7	PF3D7_1419300	Glutathione S-transferase	+
20.9	PF3D7_1020900	ADP-ribosylation factor 1	+
17.3	PF3D7_1121600	Circumsporozoite protein-related antigen	+
14.1	PF3D7_0617800	Histone H2A	+
13.9	PFC10_API0030	40S ribosomal protein S12	+
13.7	PF3D7_0503400	Cofilin/actin-depolymerizing factor homolog 1	+
11.9	PF3D7_0309600	60S acidic ribosomal protein P2	+

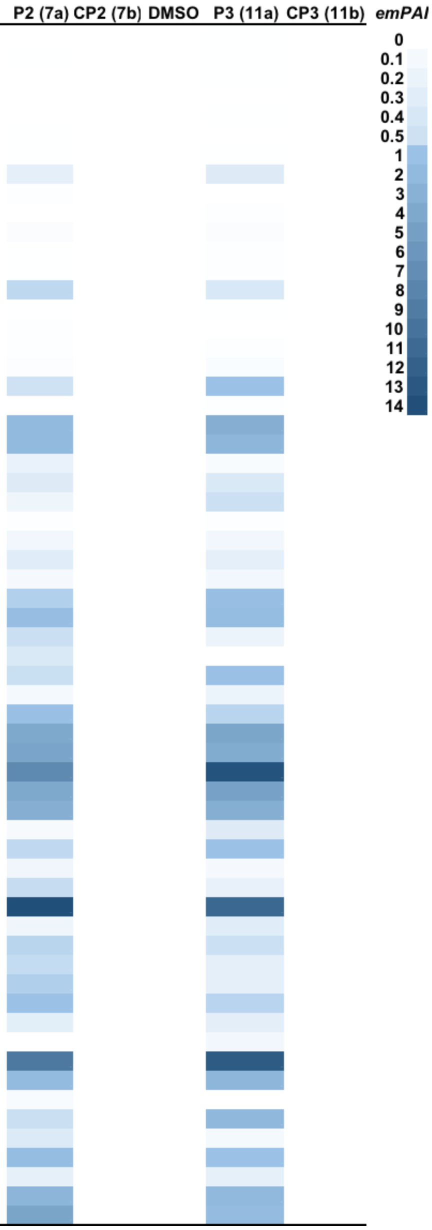

Figure S3. Heat map analysis of *P. falciparum* proteins identified with trioxolane azide probe **P2 (7a)** and artemisinin azide probe **P3 (11a)**, *vs.* controls (deoxyether analogues **CP2 (7b)** and **CP3 (11b)** and DMSO), sorted according to proteins molecular weight (KDa). The entire proteomic data set identified by **P2 (7a)**, **P3 (11a)**, **CP2 (7b)**, **CP3 (11b)** and DMSO solvent represented in heat map analysis. The analysis was carried out by plotting the average of *em*PAI values for each protein (n= 2 independent replicates in the case of **P2 (7a)**, **P3 (11a)**, **CP2 (7b)**, **CP3 (11b)** and n=4 in case of DMSO treatment). All treatments were carried out in *situ* at 1μM probe. Each independent replicate was injected into the LC-MS/MS four times to increase the accuracy of protein identification. Proteins in the heat map plot are sorted according to their molecular weight (KDa). Proteins were manually searched for in the presence (+) and absence (-) of a GSH binding motif according to the data published by Keher et al.[14] Proteins not matching in both treatments **P2 (7a)** vs. **P3 (11a)** are illustrated in table S2. The entire data set and bioinformatics analysis used to create heat map analysis are illustrated in Dataset 1 excel file.

Table S2. Proteins not matching for endoperoxide probe treatments, **P2 (7a)** *vs.* **P3 (11a)**

N	MW KDa	Gene Name	ORF identifier	Protein description	GSH binding	emPAI
				**Proteins in P2**		
1	267.96	PF3D7_0605800	RAD50	Probable DNA repair protein RAD50	-	0.01
2	174.59	PF3D7_0731500	eba-175	Erythrocyte-binding antigen 175	-	0.02
3	92.279	PF3D7_1342600	Myosin-A	Myosin-A	-	0.02
4	51.461	PF3D7_1407900	PF14_0076	Plasmepsin-1	+	0.37
5	24.7	PF3D7_1419300	GST	Glutathione S-transferase	+	0.075
				**Proteins in P3**		
1	617.39	PF3D7_1122900	PF11_0240	Dynein heavy chain-like protein PF11_0240	-	0.01
2	400.1	PF3D7_0420300	PFD0985w	AP2/ERF domain-containing protein PFD0985w	-	0.01
3	340.52	PF3D7_1252400	nul	Reticulocyte-binding protein 3	-	0.005
4	278.68	PF3D7_0318200	RPB1	DNA-directed RNA polymerase II subunit RPB1	-	0.015
5	162.25	PF3D7_0523000	PfMDR1	Multidrug resistance protein	+	0.025
6	81.485	PF3D7_1232100	PFL1545c	Chaperonin CPN60, mitochondrial	+	0.025
7	27.161	PF3D7_0209300	ISPF	2-C-methyl-D-erythritol 2,4-cyclodiphosphate synthase	-	0.14

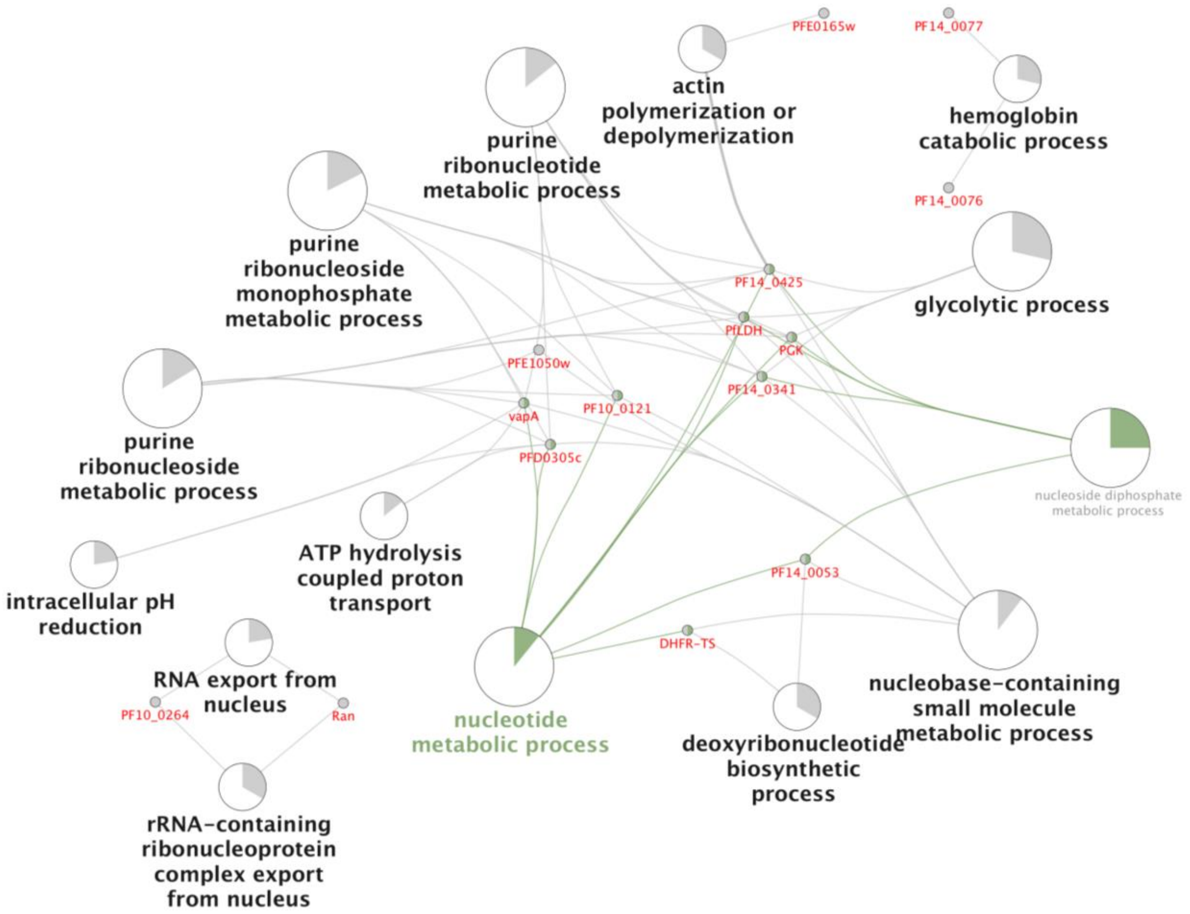

Figure S4. Gene Ontology (GO) analysis for biological pathways identified with trioxolane (**P2 (7a)**) proteome.

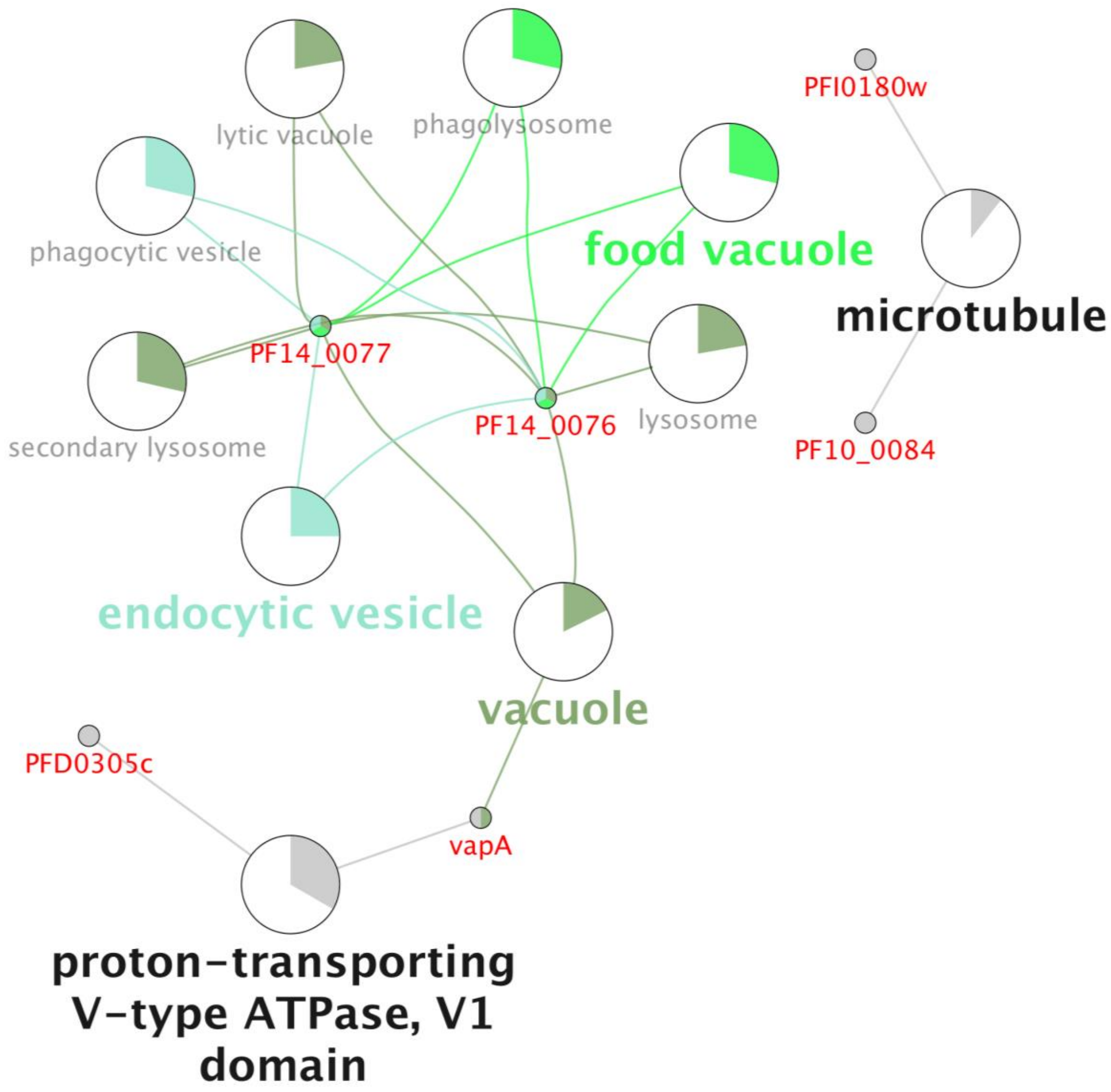

Figure S5. Cellular components targeted by **P2 (7a)** probe identifying multiple locations of proteins identified mainly in food vacuole and cytosol.

References for Supporting Information

[1] W. J. Le Noble, S. Srivastava, C. K. Cheung, *The Journal of Organic Chemistry* **1983**, *48*, 1099-1101.

[2] (a) W. Trager, J. B. Jensen, *Science* **1976**, *193*, 673-675; (b)W. Trager, J. B. Jensen, *J Parasitol* **2005**, *91*, 484-486.

[3] C. Lambros, J. P. Vanderberg, *J Parasitol* **1979**, *65*, 418-420.

[4] M. Smilkstein, N. Sriwilaijaroen, J. X. Kelly, P. Wilairat, M. Riscoe, *Antimicrob Agents Chemother* **2004**, *48*, 1803-1806.

[5] M. P. Molloy, B. R. Herbert, B. J. Walsh, M. I. Tyler, M. Traini, J. C. Sanchez, D. F. Hochstrasser, K. L. Williams, A. A. Gooley, *Electrophoresis* **1998**, *19*, 837-844.

[6] M. M. Bradford, *Anal Biochem* **1976**, *72*, 248-254.

[7] A. E. Speers, B. F. Cravatt, *Chem Biol* **2004**, *11*, 535-546.

[8] J. M. Baskin, J. A. Prescher, S. T. Laughlin, N. J. Agard, P. V. Chang, I. A. Miller, A. Lo, J. A. Codelli, C. R. Bertozzi, *Proc Natl Acad Sci U S A* **2007**, *104*, 16793-16797.

[9] A. E. Speers, B. F. Cravatt, *Curr Protoc Chem Biol* **2009**, *1*, 29-41.

[10] D. Voronin, A. F. Guimaraes, G. R. Molyneux, K. L. Johnston, L. Ford, M. J. Taylor, *Parasit Vectors* **2014**, *7*, 462.

[11] Y. Ishihama, Y. Oda, T. Tabata, T. Sato, T. Nagasu, J. Rappsilber, M. Mann, *Mol Cell Proteomics* **2005**, *4*, 1265-1272.

[12] R. Saito, M. E. Smoot, K. Ono, J. Ruscheinski, P. L. Wang, S. Lotia, A. R. Pico, G. D. Bader, T. Ideker, *Nat Methods* **2012**, *9*, 1069-1076.

[13] G. Bindea, B. Mlecnik, H. Hackl, P. Charoentong, M. Tosolini, A. Kirilovsky, W. H. Fridman, F. Pages, Z. Trajanoski, J. Galon, *Bioinformatics* **2009**, *25*, 1091-1093.

[14] S. Kehr, E. Jortzik, C. Delahunty, J. R. Yates, 3rd, S. Rahlfs, K. Becker, *Antioxid Redox Signal* **2011**, *15*, 2855-2865.
